# Strenuous physical activity, exercise, and pelvic organ prolapse: a narrative scoping review

**DOI:** 10.1007/s00192-023-05450-3

**Published:** 2023-01-24

**Authors:** Kari Bø, Sònia Anglès-Acedo, Achla Batra, Ingeborg H. Brækken, Yi Ling Chan, Cristine Homsi Jorge, Jennifer Kruger, Manisha Yadav, Chantale Dumoulin

**Affiliations:** 1 Department of Sports Medicine, The Norwegian School of Sport Sciences, Ullevål stadion, PO Box 4014, 0806 Oslo, Norway; 2 Department of Obstetrics and Gynecology, Akershus University Hospital, Lørenskog, Norway; 3 Urogynaecology Unit Hospital Clínic de Barcelona, Barcelona, Spain; 4 Department of Obstetrics & Gynaecology, VMMC & Safdarjung Hospital, New Delhi, India; 5 Kolbotn Physical Institute, Nordre Follo Municipality, Norway; 6 The Pelvic Floor Centre, Division of Surgery, Akershus University Hospital, Lørenskog, Norway; 7 Department of Obstetrics and Gynaecology, Calderdale and Huddersfield NHS Foundation Trust, Huddersfield, UK; 8 Department of Health Science Ribeirão Preto Medical School, University of São Paulo, São Paulo, Brazil; 9 Auckland Bioengineering Institute, University of Auckland, Auckland, New Zealand; 10 Paropakar Maternity and Women’s Hospital, Thapathali, Kathmandu, Nepal; 11 School of Rehabilitation, Faculty of Medicine, Université de Montréal, Montréal, Canada

**Keywords:** Exercise, Incidence, Pelvic organ prolapse, Physical activity, Prevalence, Sport

## Abstract

**Introduction and hypothesis:**

High-intensity physical activity and exercise have been listed as possible risk factors for pelvic organ prolapse (POP). The aim of the present study is to conduct a literature review on the prevalence and incidence of POP in women who engage in regular physical activity. In addition, we review the effects of a single exercise or a single session of exercise on pelvic floor support. Finally, the effect of exercises on POP in the early postpartum period is reviewed.

**Methods:**

This is a narrative scoping review. We searched PubMed and Ovid Medline, the Physiotherapy Evidence Database (PEDro), and the Cochrane Database of Systematic Reviews up to May 2022 with the following MeSH terms: “physical activity” AND “exercise” AND “pelvic floor” AND “pelvic organ prolapse”.

**Results:**

Eight prevalence studies were retrieved. Prevalence rates of symptomatic POP varied between 0 (small study within different sports) and 23% (Olympic weightlifters and power lifters). Parity was the only factor associated with POP in most studies. Three studies evaluated the pelvic floor after a single exercise or one session of exercise and found increased vaginal descent or increased POP symptoms. One prospective cohort study reported the development of POP after 6 weeks of military parashot training, and one randomized trial reported increased POP symptoms after transverse abdominal training. There is scant knowledge on exercise and POP in the postpartum period.

**Conclusions:**

Prevalence of POP in sports varies widely. Experimental and prospective studies indicate that strenuous exercise increased POP symptoms and reduced pelvic floor support.

## Introduction

Pelvic organ prolapse (POP) is a common condition in women, with reported prevalence rates varying according to whether it is reported by symptoms (1–31%), pelvic examination (10–50%), or both (20–65%) [1]. Parity and vaginal birth with pelvic floor muscle (PFM) injuries are established risk factors [2]. Handa et al. [2] reported that the strong association between POP and levator ani avulsion could be explained to a large extent by an increased levator hiatus size and weaker PFM rather than the avulsion itself. Strenuous physical work and heavy lifting have also been shown to be associated with POP [3–6]; hence, participation in strenuous sports such as weightlifting and marathon running has been suggested as a possible risk factor [7]. Physical activity is defined as “any bodily movement produced by the skeletal muscles that results in a substantial increase over the resting energy expenditure and can be performed in varying domains; as part of work/school activities, as commuting, as household chores, and as leisure activity, e.g., sport and fitness activities [8]. All four domains can include strenuous physical exertions. An exercise is one repetition of a movement, e.g. performing a sit-up, and a set of exercise is the number of times the desired number of repetitions is performed, e.g. three sets of 12 sit-ups. A session or a bout of exercise includes exercise over time, e.g. 1 h of exercise [9]. Exercise training is defined as exercise usually performed on a repeated basis over an extended period of time (weeks, months, years) with a specific external objective such as improvement of fitness, physical performance or health” [8]. Finally, fitness is the measurable outcome of physical activity or exercise training, and can be, for example, strength, endurance, flexibility or motor control (including, for example, balance) training. However, fitness can also to a large extent be inherited and physical activity and exercise training, depending on the mode of activity and dosage of training, may or may not improve fitness [8].

In 2004, Bø [10] proposed two hypotheses on the effects of physical activity and exercise training on the pelvic floor. An update on the evidence supporting each of these hypotheses was provided in 2019 by Bø and Nygaard [7]. The two opposite hypotheses on the effect of physical activity/exercise training on the pelvic floor are:

- Hypothesis 1: General exercise training strengthens the pelvic floor. The theory is that the impacts that occur during physical activity may stretch and fatigue the PFM, leading to a training effect, and/or that impacts during exercise could lead to a co-contraction of the PFM, creating an acute indirect training effect. With time, this may reduce the levator hiatus area by causing hypertrophy and shortening of the surrounding muscles, thereby lifting the pelvic floor and the internal organs into a higher pelvic location. Theoretically, such morphological changes could reduce the risk of urinary incontinence (UI), anal incontinence (AI), and POP. On the other hand, it could also theoretically negatively impact labor and childbirth; in particular, the second stage of labor.
- Hypothesis 2: general exercise training overloads, stretches, and weakens the pelvic floor. This hypothesis is based on the premise that physical activity increases intra-abdominal pressure (IAP). If the PFM are not able to co-contract quickly or strongly enough to counteract this increased pressure or withstand the ground reaction forces or, more likely, are not firm enough to maintain the location of the internal organs in an optimal position to keep the levator hiatus closed, the levator hiatus could become wider and the pelvic floor would descend. According to this theory, overload of the PFM may increase the risk of UI, AI, and POP, but on the other hand, could also result in easier childbirth [7, 10].

Although there is a huge body of studies on the prevalence of UI in women who engage in physical exercise [7, 11–13], the authors of these reviews have pointed out the sparsity of studies on the prevalence of POP in women who engage in exercise training and different sports. A scoping review [14] found only one study related to exercise and POP [15]. POP is known to negatively affect quality of life and participation in social activities [16], and a recent study among 4,556 Australian women with self-reported pelvic floor dysfunction (PFD) found that 37% reported they had stopped exercising because of POP symptoms [17]. However, a study of middle-aged women [18] found that median overall lifetime activity, expressed in the metabolic equivalent of task (MET) hours/week, did not differ significantly between women with and without POP. In adjusted analyses, there was no associations between odds of POP and overall lifetime physical activity, lifetime leisure activity, or lifetime strenuous activity. There was, however, a marginally significant nonlinear relationship between participation in strenuous physical activity (≥21 h/week) during the teenage period and an increase in the log-odds of POP later in life [18]. Other studies that have compared women with and without POP, have found that heavy lifting at work increased the likelihood of POP [19, 20].

Regular physical activity and exercise training throughout the lifespan is an important modifiable factor for prevention of illnesses and promotion of health for men and women [21]. Although less than 30% of the adult population follow the World Health Organization’s (WHO) recommendation of regular physical activity [21], in most Western societies, increasing numbers of women participate in high-intensity sports and fitness activities, often to a professional level. To follow an exercise and health promotion strategy it is important to understand whether exercise, per se, or the type or intensity of exercise, may predispose women to POP, or on the other hand have the capacity to prevent POP. The aims of the present study were therefore to investigate:

1. Prevalence and risk factors of POP among women who engage in physical activity/exercise training
2. Short-term effect of a single exercise or an exercise session on the structural support of the pelvic floor
3. Incidence of POP in women after long-term exercise
4. The effect of exercise in the early postpartum period on POP

## Materials and methods

This was a narrative scoping review [22]. A search strategy of the literature was conducted using the following MeSH terms, from PubMed (from 1946 to May 2022, Ovid Medline (from 1946 to May 2022), the Physiotherapy Evidence Database (PEDro), and the Cochrane Database of Systematic Reviews up to May 2022: “physical activity” AND “exercise” AND “pelvic floor” AND “pelvic organ prolapse”.

Inclusion criteria were full-text articles published in the English language. The reference lists of found articles were searched for additional studies. Eligible studies in the search were those answering the four research questions. For the prevalence studies we extracted the following data in a table: authors, age of publication, and country of the study; study design, description of sport or physical activity, age, and number of participants; response rate, outcome measure of POP; reported outcome on the prevalence of POP and results of the analysis of associated factors.

For incidence, short-term, single-session, and long-term prospective studies we describe the studies in the text and not in tables.

## Results

### Prevalence and risk factors of POP in exercising women

Table 1 shows the studies reporting on the prevalence of POP. Eight cross-sectional studies from Australia, Brazil, Norway, and the USA were retrieved. The studies were published between 2016 and 2021 and all studies except one [23] were conducted as web-based surveys. Number of participants varied between 163 [23] and 3,934 [15]. Most studies included both parous and nulliparous women and results were analyzed according to parity. Response rate was only available and known in one study [24]. Three studies did comparisons between athletes and controls or between different sports [23, 25, 26]. The studies covered several sports (running, CrossFit, cheerleading, power lifting, Olympic weightlifting, volleyball, artistic gymnastics, trampoline, judo, swimming, and triathlon). All studies used questions on symptoms of POP from validated questionnaires (Table 1).

**Table 1:** Prevalence of pelvic organ prolapse (POP) in physically active women and elite athletes

	Design	Population/*n*	Sport	Response rate	Outcome measure	Results
Yi et al., USA [27]	Cross-sectional study, online survey	311 English-speaking women, aged >18 on a nationwide list of triathlete groups associated with the USA Triathlon (USA Triathlon, Colorado Springs, CO, USA) organisation + a women-specific triathlon Internet forum Age: range 35–44 Nulliparous: 56.0% Primiparous: 11.1% Multiparous: 32%	Triathlon	Not reported/not available owing to online survey	EPIQ: “Do you have a sensation that there is a bulge in your vagina or that something is falling out from your vagina?”	5% reported POP symptoms 3% of those with POP were highly bothered POP was more prevalent among parous women (8% vs 3 %, *p*=0.05) No association with POP and female athlete triad (disordered eating, menstrual irregularities, and osteoporosis)
Almeida et al., Brazil [23]	Cross-sectional study, survey filled in by participants with researchers present to answer questions	67 female athletes from a large sports club in the city of Belo Horizonte, Minas Gerais State, and 96 non- athletes from a teenager health care center and from physiotherapy/ physical education graduation programs Median age: Athletes: 18.0 (IQD 5) Controls: 21 (IQD 4) Nulliparous	Amateur athletes: volleyball (*n*=23), artistic gymnastics/trampoline (*n*= 9), judo (*n*=9), swimming (*n*=26)	Not reported, but reported that not all participants answered POP questions	“Are you aware of/do you see a lump or bulge coming down in your vagina?”	Athletes: 0 of 48 Non-athletes: 2 (2.3%) of 88
Forner et al., Australia [15]	Cross-sectional study; online survey	3,934 women contacted via Facebook and other social media Mean age: 40.3 (SD 11.3) years Vaginal birth: 43.2% Parity 1: 17.2% Parity 2: 25.1% Parity 3+: 14.4% Forceps: 10.3% CS: 7%	Women who lift light (≤15 kg), moderate (16–50 kg), and heavy (>50 kg) weights for exercise, and those who do not lift weights for exercise	Not reported/not available owing to online survey	PFDI-20: “Do you usually have a bulge or something falling out that you can see or just feel in the vaginal area?”	14.4% with POP symptoms Weight lifted, age, vaginal parity, history of constipation or hemorrhoids, and family history of POP associated with symptoms Physically active women lifting weights ≤15 kg are more likely to report symptoms of POP than women lifting weights greater than 50 kg (59.7% vs 15.2%; adjusted OR 2.1; 95% CI 1.7–3.4) No relationship between POP symptoms and BMI, forceps delivery, CS, hysterectomy, or menopausal status
High et al., USA [28]	Cross-sectional, study, online survey	314 women from affiliate gyms in the USA. Lists were obtained from the official website of CrossFit, Inc, Facebook Distributed to 990 CrossFit gym owners Mean age: 36 (SD 10) years Nulliparous: 32% >1 vaginal delivery: 47% CS: 21%	Cross-fit	Not reported/not available owing to online survey	PFDI-20: “Do you usually have a bulge or something falling out that you can see or just feel in the vaginal area?”	3.2% with POP symptoms POP associated with a higher reported number of vaginal births and higher overall parity, but not age or BMI
Skaug et al. Norway [24]	Cross-sectional study, online survey	180 women aged >18 years and participation in at least one Norwegian National Championship (NCC) in powerlifting or Olympic weightlifting during the 2018/2019 season Mean age 31 (SD 10.7) 27.2% parous Parity: 2.2 (SD 1.3, range 1–9)	Power lifters and Olympic weightlifters based on list of participants in Norwegian championships	54.1%	ICIQ-VS: “The complaint of a bulge” or “something coming down toward or through the vaginal introitus”	23% with POP symptoms Mean score of bother (0–10) was 1.0 (SD: 2.1, range: 0–8) and 1.0 (SD: 1.6, range: 0–3) for bulge felt on the inside and outside of the vagina respectively Four (10.8%) with symptoms of a bulge felt on the inside scored ≥5 Occasional and daily straining on voiding were positively and chronic disease negatively associated with POP
Carvalho et al., Brazil [26]	Cross-sectional comparison study, online survey	156 normal weight women (78 cheerleaders and 76 non-athletes). Non-athletes recruited from the graduate program of physiotherapy, nursing, and physical education Mean age 20.8 (SD 2.3) years Nulliparous	Cheerleaders	Not reported/ not available owing to online survey	ICIQ-VS “Do you feel a lump or bulge coming out of your vagina completely, so that you can feel it or see it on the outside?”	9 women (6 cheerleaders (7.7%) and 3 non-athletes (3.9%) reported having POP symptoms (*p*=0.32) Bases: 1 (2.6%); flyers: 5 (12.5%)
Pisani et al., Brazil [38]	Cross- sectional study, online survey	828 women from gyms affiliated to the CrossFit brand Mean age: 30.6 (SD 6.6) years Nulliparous: 74.4% Primiparous: 14.5% Multiparous: 11.1%	CrossFit	Not reported/not available owing to online survey	ICIQ-VS: “Do you feel a lump or bulge coming out of your vagina completely, so that you can feel it or see it on the outside?”	1.4% with POP symptoms No association with any of the anthropometric variables. POP was not related to CrossFit training (frequency, experience)
Forner et al., Australia [25]	Cross-sectional study, online survey	1,379 women (521 runners, 858 CrossFit) recruited through social media (Facebook, Instagram and Twitter) Mean age: Runners: 38.4 (SD: 9.2) years CrossFit: 38.5 (SD 8.8) years Ns between groups Vaginally parous: Runners: 56.6% CrossFit: 52.7% Ns between groups. Ns difference in parity	Runners, CrossFit participants	Not reported/ not available owing to online survey	PFDI-20: “Do you usually have a bulge or something falling out that you can see or just feel in the vaginal area?” POPDI-6: Question above from PFDI-20	Runners: 12.7% CrossFit: 7.8%, *p*=0.003 Vaginally parous runners reported more symptoms of POP (19.0% vs 12.2%, *p*=0.023)

*CS* cesarean section, *BMI* body mass index, *EPIQ* Epidemiology of Prolapse and Incontinence Questionnaire, *ICIQ-VS* International Consultation on Incontinence Questionnaire—Vaginal Symptoms, *IQD* interquartile distance, *Ns* nonsignificant, *PFDI* Pelvic Floor Distress Inventory, *PFIQ* Pelvic Floor Impact Questionnaire, *POPDI-6* Pelvic Organ Prolapse Subscale

Prevalence of symptomatic POP varied between 0 in small samples of different sports [23] and 23% in power lifters and Olympic weightlifters [24] (Table 1). Our search retrieved no studies on marathon runners, but two studies investigated prevalence among runners [25, 27]. Forner et al. [25] found a slightly but statistically significant higher prevalence in runners of 12% vs 7% among CrossFit participants, whereas Yi et al. [27] found a prevalence of 5% among triathletes (Table 1). No statistically significant differences in prevalence were found in two studies comparing athletes with non-athletes [23, 26]. One study comparing women lifting heavy, moderate, and light weights vs no weight found that the proportion of participants with symptoms of POP in the heavy-lifting group (7.1%) was significantly lower than both the inactive group (21.3%) and light-lifting group (19.4%, *p*<0.001) [15].

Table 1 also shows the results of studies of associations of POP and other factors. Parity was associated with symptoms of POP in four studies [15, 25, 27, 28], but not in the study on Olympic weightlifters and power lifters. In that study the only statistically significant association was straining on voiding [24]. The Female Athlete triad (disordered eating, menstrual irregularities, and osteoporosis), BMI and age were not associated with POP in any of the studies investigating these factors (Table 1).

Three studies compared different pelvic floor muscle variables such as strength, endurance, and resting activity or pelvic floor support between exercisers and controls. Braekken et al. [19] compared 49 middle-aged women with POP stage ≥2 with 49 women with POP stage 0–1. They found that PFM strength (OR 7.5; 95% CI 1.5–36.4) and PFM endurance (OR 11.5; 95% CI 2.0–66.9), measured by manometry, were independently related to POP. Heavy occupational work was associated with POP, but low-intensity physical activity was not.

Machado et al. [29] used vaginal palpation and found no differences in ability to contract the PFM between nulliparous and healthy women performing CrossFit and non-exercisers. Further, there was no difference in resting tone, maximum voluntary contraction, fast contractions and sustained contraction tested by surface electromyography (sEMG). Middlekauff et al. [30] compared PFM resting pressure and strength in 35 nulliparous CrossFit participants and 35 nulliparous women who walked for fitness, using manometry and POP-Q. They found no significant differences in PFM resting pressure, PFM strength or vaginal descent.

### Can a single exercise or one exercise session cause weakness to the pelvic floor muscles or decreased pelvic floor support?

We retrieved three studies on this research question. Ree et al. [31], in a prospective cross-over study, found a reduction of 17% in PFM strength as measured by manometry after one 90-min bout of strenuous running, jumping, and weightlifting exercise compared with 90 min of rest with no exercise. POP signs and symptoms were not assessed in this study.

Ali-Ross et al. [32] included 54 women the day before POP surgery and asked them to stay mostly active between 4 and 6 h and then to participate in a 1-h bout of structured physical activity. The prescribed activities involved walking about for 45 min (including going up and down one flight of stairs), standing up from sitting five times, bending down as if to pick something off the floor ten times and jogging/stamping briskly on the spot for 1 min. POP-Q, Pelvic Floor Distress Inventory (PFDI), and Pelvic Floor Impact Questionnaire (PFIQ) were used for assessment before the exercise and after a night’s sleep at the hospital. An increased severity of POP was found on POP-Q examination following physical activity with a significant increase in POP-Q stage in five vaginal parameters (Aa, Ba, C, Ap, and Bp; *p*<0.001), but this change in POP signs was not associated with worsening of POP symptoms and greater impairment of quality of life. There was no non-exercising control group in this study.

Middlekauff et al. [30] compared 35 healthy nulliparous CrossFit-practicing women with 35 recreationally active women, mean age 24.8 (± 4.3) years before and after one 25-min bout of CrossFit exercise or walking. They found increased maximal vaginal descent assessed by ultrasound and decreased vaginal resting pressure (measured by manometry) at rest and on PFM contraction in both groups. There was no significant difference in PFM strength between the groups.

### Incidence of POP after long-term physical activity

We found two studies investigating this research question. One prospective study [33] investigated the change in pelvic organ support by POP-Q in 116 women who attended a 6-week summer military training program in the USA. They found that among the 37 women participating in paratroop training, there was a significant increase in the likelihood of developing stage II POP (RR=2.72, 1.37 < RR <5.40; *p*=0.003) compared with a control group. Post-test the examiner was blinded to the results at pre-test. At the POP-Q examination before the training 52%, 46%, and 2% had POP stage 0, 1, and 2 respectively. At the examination after the 6-week paratrooper training 24%, 54%, and 22% had POP stage 0, 1, and 2 respectively.

Brandt and Janse Van Vuuren [34] conducted a 6-month double-blind RCT including 81 women aged 18–75 years with POP stage I–III scheduled for reconstructive POP surgery. All women had surgery but were randomized to either PFMT (*n*=24 analyzed), PFMT with transversus abdominal training (*n*=28 analyzed) or usual care (*n*=29 analyzed) after the operation. The rationale for including transversus abdominal training was that this may cause co-contraction of the PFM and thereby is assumed to improve outcome. One surgeon with >10 years’ experience conducted all surgeries and there were no statistically significant differences in type of surgery between the randomized groups. PFMT included 12 appointments with a physical therapist with assessment of the PFM using observation, palpation, ultrasound, and sEMG. The participants were exercising five times per week with progression of the number of contractions to 10 and holding time to 10 s. Performance of the exercises started in supine and ended up in standing positions. Exercises were patient specific with individual progression based on the evaluation of PFM function. The abdominal training included co-contraction of the transversus abdominus, PFM, and multifidus muscles ten times for 10 s each. Performance of correct abdominal contraction was assessed by a pressure biofeedback unit. There was a progression from lying to standing positions until the same holding times were reached. Further progression included adding low-load limb movement, introducing unstable surfaces, and eventually introducing global stability and strengthening exercises. Usual care included instruction to precontract the PFM during increase in IAP with no further follow-up. At 6 months the group doing transversus abdominal training in addition to PFMT had a statistically significant increase in bulging and discomfort on the Prolapse Quality of Life questionnaire.

### Physical activity, exercise training, and POP during the postpartum period

We found two prospective studies investigating the association between exercise habits post-partum and PFDs at 1 year after birth [35, 36]. Both studies assessed nulliparous women in their first pregnancy and followed them through to 12 months postpartum. Tennfjord et al. [35] found that women with physically strenuous occupations such as walking and/or standing >50% of the working day (79 of 177 (45%)) and daily heavy lifting >10 to 20 times a day (16 of 177 (9%)), were more likely to report POP symptoms 12 months postpartum (OR = 3.0 [95% CI = 1.2 to 7.3]). Nygaard et al. [36] reported a marginally, but statistically significantly higher prevalence of women with worse POP-Q score in the moderate exercise training group, but otherwise neither study found significant associations between early resumption of either low or high impact exercise and symptoms of POP or other PFD. The results of pelvic floor support by POP-Q were dichotomized as maximal vaginal descent <0 cm (better support) versus ≥0 cm (worse support). Pelvic floor symptom burden was considered positive with reports of ≥1 bothersome symptom in ≥2 of 6 domains, assessed using the Epidemiology of Prolapse and Incontinence Questionnaire [36]. In another publication on the same study group, Nygaard et al. [37] not only found no association between pelvic floor support and IAP and other measures of pelvic floor loading, but no protective effect of greater muscular fitness either.

## Discussion

Compared with the literature on physical activity and exercise training and UI, there is scant research data on the impact of physical activity or specific exercises/one session of exercise on POP signs and symptoms and pelvic floor support. In this review we included studies on both prevalence and incidence (short and long term) in addition to studies in the postpartum period. We found that prevalence of POP symptoms varied between 0 and 23% in studies of different sports with the highest prevalence found among women participating in Olympic weightlifting and power lifting. Most studies included both nulliparous and parous women and all but one study found that parity was associated with POP among exercising women. Further, results of the few prospective studies indicated that strenuous short- or longer-term physical activity/exercise may negatively affect pelvic floor support. There is scant knowledge on the impact of exercise on pelvic floor support and POP in the postpartum period.

### Prevalence of POP in exercising women

The prevalence studies applied quite similar questions of symptoms of POP, either as recommended single questions (feeling of a bulge) or as part of validated questionnaires encompassing multiple POP symptoms. Hence, the definition of the condition may not explain the differences in prevalence between studies. As there are few studies, those available only cover a small number of sports. From a theoretical standpoint, both long distance running/marathon running, and heavy weightlifting could potentially negatively influence the pelvic floor owing to the impact from repeated ground reaction forces and increased IAP. Only two studies investigated prevalence among runners [25, 27]. Forner et al. [25] found a slightly but statistically significantly higher prevalence in runners (12.7%) than CrossFit participants (7.8%), whereas Yi et al. [27] investigated triathletes and found a prevalence of 5%. Triathletes also run long distances in addition to the other elements of swimming and bicycling, but since both triathlon and CrossFit also include other modalities, there is a need for more prevalence studies among long distance and marathon runners. The highest prevalence was found in Olympic weightlifters and power lifters at 23% [24]. CrossFit participants also lift heavy weights but the prevalence in three studies investigating CrossFit participants reported lower prevalence (between 1.4% and 7.8%) [25, 28, 38]. Both factors, age and parity, were similar in these studies and cannot explain the difference in prevalence. However, there are differences between the participants of CrossFit studies and the Olympic weightlifters and power lifters. Skaug et al. [24] included only high-level competitive athletes. Gephart et al. [39] assessed IAP during 10 repetitions of 13 different CrossFit exercises in 5 experienced and active CrossFit exercisers and 5 women who were not regularly engaged in CrossFit. Parous women (*n*=5) achieved higher IAP with pushups, whereas nulliparous women (*n*=5) achieved higher IAP when performing jump rope, thrusters, and wall balls. Experience with CrossFit did not affect mean peak IAP achieved with exercise. Interestingly, it was noted that in back squats there was a significant increase in IAP as participants progressed through repetitions. In sit-ups the IAP was decreasing. The findings of this study need further investigation in larger samples of both nulli- and multiparous women.

The limitation of cross-sectional studies is that symptomatic woman may restrict high-intensity exercise participation, which may lead to a selection bias. This may have been the case with the largest study so far, where Forner et al. [15] reported that fewer women exercising with heavier weights reported POP symptoms than those lifting light or no weights. Owing to the study methodology of the prevalence studies with use of social media as the recruitment platform with no knowledge of the total population, the response rate is not reported in most of the studies. This makes generalization impossible, and the studies may be flawed with selection bias.

### Incidence and influence of single exercise, bouts of exercise, and long-term exercises on the pelvic floor

We only found one prospective study following women from start to cessation of a 6-week strenuous training program [33]. The study showed that there was an increase in POP signs assessed by POP-Q in a group of parachute jumpers after a summer training program. This finding is supported by some of the short-term studies assessing PFM resting activity and strength and pelvic floor support immediately after one session of strenuous physical activity [30–32], where PFM strength and support were reduced. In a systematic review investigating the increase in IAP during different high-intensity resistance exercises, Blazek et al. [40] found that the highest IAP was recorded during squats (over 200 mmHg) followed by deadlift, slide row, and leg press (161–176 mmHg), and the lowest IAP was found during bench press (79±44 mmHg). We have no information from Larsen and Yavorek [33] about the content of the summer military camp program but may assume that some of the above-mentioned exercises with high IAP were included in addition to the actual parachute jumping. In general, further studies on the impact of different exercises on pelvic floor support are warranted. Future studies also need to include more information on the actual mode and dosage of the exercises performed.

The finding of the RCT that women performing transverse abdominal exercises in addition to PFMT had more POP symptoms than women performing only PFMT is new and interesting [34]. Increase in IAP during abdominal exercise has been hypothesized to have a negative impact on pelvic organ support [41, 42]. However, there are short experimental studies that do not support the hypothesis that abdominal exercises could have this effect. In an overview of short-term exercises Bø and Nygaard [7] reported variable increases in IAP during curl-up between 7 and 100 cmH_2_0, sit-up between 14 and 133 cmH_2_O and the plank between 23 and 95 cm H_2_O from different studies. Interestingly, Weir et al. [43] measured IAP during different exercises in 30 women and concluded that abdominal crunches, climbing stairs, walking on a treadmill, and many lifting activities did not increase IAP significantly more than standing up from a chair. This was confirmed by the study of O’Dell et al. [41]. Also, Tian et al. [44] found that performing what physical therapists advocated as a recommended "pelvic floor-safe" version of exercises did not necessarily protect the pelvic floor. However, the latter study was conducted in asymptomatic women, and it would be interesting to repeat the experiment in a group of women with POP symptoms. Based on results from studies included in the present review, the highest prevalence of POP was reported among power lifters and Olympic weightlifters [24]. This indicates that heavy lifting, which increases IAP substantially, may be the most important risk factor for POP. However, there are many factors that may influence IAP such as difference in performance of exercise and errors that may occur when assessing IAP. Results should therefore be considered with caution. A short-term experimental study comparing sEMG response before and after expected and unexpected perturbations in women with and without UI found that women with UI had increased PFM and abdominal activation. This is challenging the belief that incontinence is associated with reduced muscle activity [45]. However, the study did not include women with POP, and more experimental studies are needed to understand the influence of heavy lifting and abdominal training on PFM response in women with POP.

As some studies show that there may be a negative effect on pelvic floor support after shorter-term exercise, there may be a risk that longer-term repeated strenuous exercise may be a risk factor for development of POP as was shown by Larsen and Yavorek [33]. There is an urgent need for more prospective studies to test this hypothesis. Importantly, Shaw et al. [42] registered variability in increase in IAP amongst individuals doing the same activity, especially in activities that required regulation of effort. This may explain why some women are at risk for development of POP during exercise and others are not. One could assume that some women with inherited risk factors such as weak connective tissue and increased flexibility/laxity, e.g. benign hypermobility joint syndrome [46], would be more exposed to the development of POP due to strenuous exercise than others. This may also apply to those with weaker PFM and levator ani muscle trauma. These hypotheses need further investigation. The limitations of the experimental and long-term studies of physical activity on the pelvic floor are that few studies have reported measurement of the PFM, IAP, and pelvic floor support, and that there is a lack of reliable and valid measurement methods during physical activity [47].

### The postpartum period

Today, many female athletes are at the peak of their sporting career in their 30s and want to combine motherhood with continued participation in high-level competition [48]. The very important question is when it is safe to start with strenuous exercise in the postpartum period. So far there is little scientific evidence to guide progression of strenuous exercise after childbirth [9]. Recovery of the levator hiatus area, a reflection of recovery of the levator ani muscle and associated connective tissue and nerves, is generally maximized at 4–6 months postpartum [49, 50]. Bladder neck mobility increases after vaginal birth and, although the support to the bladder neck improves postpartum, mobility remains higher than when measured at 37 weeks’ gestation [49, 51]. However, this depends on the degree of peripheral nerve, connective tissue, pelvic floor muscle, and perineal injury during birth and hormonal influence during pregnancy. The impact of these factors on the development of POP in the early postpartum period needs further study, as does the influence of early PFM training. The two studies found in our review of exercise and strenuous work with POP in the postpartum period reported some negative effects on development of POP, but they also reported that the number of women participating in high-intensity exercise was low, which potentially influenced the ability to draw any firm conclusions. Nygaard et al. [36] found no association between levator ani muscle defects, physical activity, and PFDs; however, defects were not assessed clinically, but rather estimated based on known delivery risk factors. Moore et al. [52] asked 881 postpartum women about return to running. They found that median time to first postpartum run was 12 weeks. Running during pregnancy (OR: 2.81 (1.90 to 4.15)), a high weekly running volume (OR: 1.79 (1.22 to 2.63)), lower fear of movement (OR: 0.53 (0.43 to 0.64)), and not suffering vaginal heaviness (OR: 0.52 (0.35–0.76)) increased the odds of return to running. There is an urgent need for more basic research on the influence of strenuous work and exercise within the early postpartum period (from childbirth and up to 6 months). The role of inciting factors such as birth-related PFM injuries needs further prospective studies with clinical assessment before clear conclusions can be drawn about the potential harm or benefit of high-impact exercise on the prevalence of POP. More than 50% reduction in PFM strength and endurance has been found 6 weeks postpartum compared with pregnancy values [53]. PFM strength and endurance in women with major levator ani tears are further reduced by 50% compared with women with no tears [54].

As most of the research to date is in the form of cross-sectional studies, inferring causation from these studies is not possible. It is also clear that responsive, reliable, and valid methods of assessment of the PFM, either during exercise or after a bout of exercise, is difficult [47]. If high IAP is suspected to be a potential cause of the development of POP during exercises such as Olympic weightlifting, then it is imperative that measuring IAP and the ability of the PFM to respond to that is as accurate as possible. Thus, the evidence from the reported studies on prevalence and incidence of POP symptoms is still scant and equivocal, as is the effect of different single exercises on PFM variables and pelvic floor morphology. Whether the short-term signs of fatigue found in some studies may lead to later hypertrophy of the PFM and whether there is a difference in response between nulliparous and parous women are interesting questions for future studies. There is also a need for more experimental studies in the postpartum period addressing the impact of different exercises on the pelvic floor in this important time of PFM healing. The importance of assessment of the PFM has recently been included in recommendations for follow-up surveillance before return to sport after childbirth [52]. Limitations with studies during pregnancy and in the postpartum period are the lack of knowledge of physical activity level and also POP and pelvic floor support before pregnancy.

Although strenuous exercise may have a negative effect of pelvic floor support in some women, regular endurance and strength training are important factors for women’s health [21]. The authors of this review strongly support WHO recommendations for increasing physical activity level among adults including both moderate-intensity aerobic activity and muscle-strengthening activities for the major muscle groups of the body [21], and would not recommend any women to stop exercise because of PFD. However, they may be recommended to start with low-impact exercises such as walking, swimming, and bicycling (no running and jumping) while they are conducting a PFM training program.

Pelvic floor muscle training has level 1/recommendation A to be effective in the treatment of UI and POP [55–57]. We have not found any RCTs or studies using other designs investigating the effect of PFMT on POP in women who engage in regular physical exercise or those who engage in heavy lifting or strenuous physical work. Many studies have shown that girls and women who engage in physical exercise and athletes are not aware of their pelvic floor and do not know how to exercise the PFM [24, 58, 59]. PFMT, conducting a voluntary contraction both before and during an increase in IAP (termed “the knack”) [60, 61], and strength training over time [57, 62], have the potential to prevent and treat POP in women performing strenuous exercise. The immediate effect of “the knack” during exercise and the effect of PFMT over time in women performing strenuous exercise training and sports needs further investigation.

Strengths of the present review is the comprehensive search including cross-sectional, short-term experimental, and prospective studies. The review concentrates on POP only and is aimed at filling, and pointing out, knowledge gaps on the influence of strenuous physical activity for this condition. POP is the PFD where the effect of physical activity has caused most concern and where other authors have recommended further focus to guide future studies. Limitations are the limited number of published studies and within a relatively small number of sports. In addition, only a few research groups have assessed pelvic floor anatomy, IAP, and PFM response during activity and variables such as resting tone, strength, and endurance. There are few studies comparing physical activity with rest or different activities and few controlled prospective studies. With the few high-quality studies available, we argue that a narrative review is the best format with which to address the current knowledge and point out knowledge gaps for future trials. Owing to inclusion of studies of different designs, we did not perform a quality assessment of the study methodology. Most of the studies are cross-sectional and inferring causation from these studies is not possible.

## Conclusion

Prevalence of POP symptoms varies between studies and between sports. The highest prevalence of 23% was found between female Olympic weightlifters and power lifters. Short-term experimental studies and studies following exercisers prospectively find that strenuous exercise may negatively impact pelvic floor support. There is a shortage of studies on the effect of general physical activity and exercise training on the pelvic floor in the postpartum period. Further studies on the prevention of POP among women performing strenuous physical activity and exercise training are warranted.
